# CD4^+^CD25^hi^FOXP3^+^ Regulatory T Cells and Cytokine Responses in Human Schistosomiasis before and after Treatment with Praziquantel

**DOI:** 10.1371/journal.pntd.0003995

**Published:** 2015-08-20

**Authors:** Yvonne Schmiedel, Ghyslain Mombo-Ngoma, Lucja A. Labuda, Jacqueline J. Janse, Brechje de Gier, Ayôla A. Adegnika, Saadou Issifou, Peter G. Kremsner, Hermelijn H. Smits, Maria Yazdanbakhsh

**Affiliations:** 1 Centre de Recherches Médicales de Lambaréné (CERMEL), BP118 Lambaréné, Gabon; 2 Institut für Tropenmedizin, Universität Tübingen, Tübingen, Germany; 3 Departement de Parasitologie-Mycologie, Faculté de Médecine, Université des Sciences de la Santé, BP 4009, Libreville, Gabon; 4 Department of Parasitology, Leiden University Medical Center, Leiden, The Netherlands; George Washington University, UNITED STATES

## Abstract

**Background:**

Chronic schistosomiasis is associated with T cell hypo-responsiveness and immunoregulatory mechanisms, including induction of regulatory T cells (Tregs). However, little is known about Treg functional capacity during human *Schistosoma haematobium* infection.

**Methodology:**

CD4^+^CD25^hi^FOXP3^+^ cells were characterized by flow cytometry and their function assessed by analysing total and Treg-depleted PBMC responses to schistosomal adult worm antigen (AWA), soluable egg antigen (SEA) and Bacillus Calmette-Guérin (BCG) in *S*. *haematobium*-infected Gabonese children before and 6 weeks after anthelmintic treatment. Cytokines responses (IFN-γ, IL-5, IL-10, IL-13, IL-17 and TNF) were integrated using Principal Component Analysis (PCA). Proliferation was measured by CFSE.

**Principal Findings:**

*S*. *haematobium* infection was associated with increased Treg frequencies, which decreased post-treatment. Cytokine responses clustered into two principal components reflecting regulatory and Th2-polarized (PC1) and pro-inflammatory and Th1-polarized (PC2) cytokine responses; both components increased post-treatment. Treg depletion resulted in increased PC1 and PC2 at both time-points. Proliferation on the other hand, showed no significant difference from pre- to post-treatment. Treg depletion resulted mostly in increased proliferative responses at the pre-treatment time-point only.

**Conclusions:**

*Schistosoma*-associated CD4^+^CD25^hi^FOXP3^+^Tregs exert a suppressive effect on both proliferation and cytokine production. Although Treg frequency decreases after praziquantel treatment, their suppressive capacity remains unaltered when considering cytokine production whereas their influence on proliferation weakens with treatment.

## Author Summary

Schistosomiasis, a parasitic worm infection, affects over 240 million people worldwide, especially children in sub-Saharan Africa. It is associated with immune hypo-responsiveness which results in an inability of the immune system to eliminate parasites. Animal models suggest that helminths induce regulatory T cells (Treg) which suppress effector cells and dampen anti-parasite activity as part of the parasites’ own strategy for survival in the human host. However, little is known about the functional capacity of Tregs during human *Schistosoma haematobium* infection and their interaction with adaptive responses. We designed a longitudinal study addressing the question of how anti-parasite treatment influences effector T cell activity and Treg function in peripheral blood of schoolchildren living in an *S*. *haematobium* endemic area in Lambaréné, Gabon. Our findings show that schistosome infection is associated with increased Treg frequency and that Tregs exert a suppressive effect on immune cell function in terms of both proliferation and cytokine production. Although Treg frequency decreases after anti-schistosome treatment, their suppressive capacity remains unaltered for cytokine production but their influence on proliferation weakens with treatment. By understanding how immune system is prevented from killing parasites, we hope to offer a novel route for intervention to achieve an immunological cure.

## Introduction

The immune system has evolved several regulatory mechanisms to maintain immune homeostasis, prevent autoimmunity and restrain inflammation [1–3]. Many pathogens have developed mechanisms to manipulate the regulatory network of the host to their advantage, thereby generating conditions that ensure their survival for a prolonged period of time. In particular FOXP3^+^ regulatory T (Treg) cells have been shown to play a major role in the control of various parasitic infections suppressing local tissue damage and pathology that would result from otherwise over-reactivity. However, enhanced Treg cell activity may also allow the long-term survival of the parasite as the host is hampered from fighting the intruding pathogen effectively [4].

Schistosomiasis is a helminth infection affecting over 240 million people worldwide, especially children [5]. When chronic in nature it has been shown to be associated with general T cell hypo-responsiveness—evident from down-modulated antigen-specific Th1 and Th2 cell responses [6,7]]. This might result from mechanisms involving peripheral anergy and suppression triggered by regulatory cells, such as Tregs [8]. For example, in experimental murine models, it was observed that the presence of regulatory T cells was associated with suppressed development of pathology [9], and down-modulated Th1 and Th2 responses [10,11], promoting parasite survival within the host [12,13]. Evidence for Treg activity in human helminth infections has been provided by the detection of T cells with a regulatory phenotype in patients with lymphatic filariasis [14], onchocerciasis [15,16] and schistosomiasis [17,18].

Effective chemotherapy with praziquantel has been shown to result in elevated antigen-specific proliferation and cytokine production, in particular interleukin (IL)-4, IL-5, and interferon (IFN)-γ [6,19–22]. Although, the frequency of Tregs, defined phenotypically as CD4^+^CD25^hi^ without considering FOXP3 as a marker, decreased substantially after treatment with praziquantel [18], their functional activity has not been studied before.

The aim of this study was to assess whether *S*. *haematobium* infection in Gabonese children was associated with induction of regulatory T cells and to evaluate Treg activity during infection. To this end immune responses were evaluated before and 6 weeks after praziquantel treatment. Moreover, the functional activity of Tregs was assessed by comparing responses before and after their removal from peripheral blood mononuclear cells by *in vitro* magnetic depletion.

## Materials and Methods

### Ethics statement

Ethical approval for the study was obtained from the Comité d’Ethique Régional Indépendant de Lambaréné. A signed informed consent form was obtained from parents or legal guardians of all children participating in the study.

### Study design

The longitudinal study was conducted at Centre de Recherches Médicales de Lambaréné (CERMEL; formerly Medical Research Unit (MRU) of the Albert Schweitzer hospital). Participants were schoolchildren from a rural area (PK15) highly endemic for *S*. *haematobium* approximately 15 km south of Lambaréné in the province of Moyen-Ogooué, Gabon [22,23]. Children were excluded from the study if they received anthelminthic treatment within the previous six weeks or had fever or other symptoms of acute illness. Fifty-two schoolchildren were recruited to participate in the study and of those twenty-eight (mean age: 10.3y (SD: 2.2); sex ratio: 15f/13m; median egg count/10ml urine: 72.5 (IQR: 24.5–296.3) pre-treatment and 0 (IQR: 0–1.5) at post-treatment; haemoglobin level: 11.1 g/dL (SD: 1.0) pre-treatment and 11.1 g/dL (SD: 1.0) post-treatment; and white blood cell level: 8.8 x10^3^/mm^3^ (SD: 3.2) pre-treatment and 9.4 x10^3^/mm^3^ (SD: 2.9)) were included in the cellular analyses. Of the 24 schoolchildren that were not included in the final analysis, 4 did not return for post-treatment visit, 5 had less than 90% clearance of *Schistosoma* eggs at 2nd treatment and in 8 donors at pre-treatment and 11 donors at post-treatment Treg depletion failed. There was no significant difference between the schoolchildren that were included in the final analysis and those that were not. To compare the frequency of CD25^hi^FOXP3^+^ Treg cells between *S*. *haematobium* infected and uninfected schoolchildren an additional 10 *S*. *haematobium*-infected participants (mean age: 12.5y (SD: 1.5); sex ratio: 9f/1m; median egg count/10ml urine: 19.5 (IQR: 3.3–216.5); haemoglobin level: 11.4 g/dL (SD: 0.31); and white blood cell level: 8.8 x10^3^/mm^3^ (SD: 0.6)) were recruited from the same rural area and seven uninfected subjects (mean age: 12.9y (SD: 2.6); sex ratio: 4f/3m; median egg count/10ml urine: 0; haemoglobin level: 10.9 g/dL (SD: 0.7); and white blood cell level: 6.3 x10^3^/mm^3^ (SD: 0.7)) were recruited from semi-urban Lambaréné. In addition to their positivity for *S*. *haematobium* infection, rural children also had significantly higher white blood cell levels (p<0.05) compared to children from semi-urban Lambaréné. When comparing the 2 cohorts, while the additional cohort of children was a little older (12.7y vs 10.3y; p<0.05), there was no significant difference in haemoglobin and white blood cell levels nor in infection intensity between the 2 groups.

### Determination of *S*. *haematobium* infection

A midstream urine sample was collected during the day and 10 ml were passed through a 12.0 μm polyamide N filter (Millipore) for the detection of *S*. *haematobium* eggs by microscopy. Children were classified as infected if at least one *S*. *haematobium* egg was detected in the urine.

### Praziquantel treatment

An initial treatment with praziquantel (40 mg/kg) was administered to *Schistosoma*-infected children at inclusion, and repeated after three weeks, in order to ensure clearance of parasites. Six weeks after initial treatment the efficacy of praziquantel was assessed by measuring egg load in urine. Donors were excluded from analysis, if their reduction in egg load was less than 90% following second treatment. All subjects who were egg-positive after the second treatment were given a third dose of treatment.

### Cell isolation and Treg depletion

Peripheral blood mononuclear cells (PBMCs) were purified from heparinized venous blood (7-10ml) by Ficoll-Hypaque centrifugation (Amersham Biosciences, Netherlands). Depletion of CD25^hi^ T cells was performed using a suboptimal concentration of CD25 microbeads (Miltenyi Biotec, Bergisch Gladbach, Germany) according to the manufacturer’s instructions. This method has been shown by us in other studies to successfully deplete FOXP3 Tregs [24]. Similar results were obtained in Gabon as shown in S1A Fig.

### PBMC culture for proliferation and cytokine production

To analyse proliferation green-fluorescent dye carboxyfluorescein succinimidyl ester (CFSE; Sigma-Aldrich, CA, USA) was used; CFSE divides over daughter cells upon cell division and can be tracked by decreasing fluorescence intensity. CD25^hi^-depleted and total PBMCs were stained with 2μM CFSE for 15 minutes at room temperature prior to culture. After labelling, cells were cultured in RPMI 1640 (Gibco, Invitrogen®, Carlsbad, CA, USA), supplemented with 10% fetal bovine serum (FBS; Greiner Bio-One GmbH, Frickenhausen, Germany), 100 U/ml penicillin (Astellas, Tokyo, Japan), 10 μg/ml streptomycin, 1 mM pyruvate and 2 mM L-glutamine (all from Sigma-Aldrich, CA, USA). Cells were stimulated in 96-well round bottom plates (Nunc, Roskilde, Denmark) with medium, 10 μg/ml AWA, 10 μg/ml SEA, or 10 μg/ml BCG (Bacille Calmette-Guérin; SSI, Copenhagen, Denmark) and incubated in 5% CO_2_ at 37.5°C. After four days, supernatants were collected and stored at -80°C, while cells were harvested, fixed with 2% formaldehyde (Sigma-Aldrich, CA, USA) and, subsequently, frozen in RPMI 1640 medium supplemented with 20% FCS and 10% dimethyl sulfoxide (DMSO; Merck KGaA, Darmstadt, Germany) and stored at -80°C.

### Flow cytometry analysis (FACS)

After thawing, CFSE-labelled cells were incubated with CD4-PE (SK3; BD Bioscience, San Diego, CA, USA) and CD25-APC (M-A251; BD Bioscience, San Diego, CA, USA), acquired on a FACSCalibur flow cytometer (BD Biosciences, San Diego, CA, USA) and data were analysed in a FlowJo Proliferation application (Tree Star Inc., Ashland, OR, US) by calculation of the fraction of CD4^+^CD25^hi^ cells that had divided from the starting population (division index). To assess Treg depletion, ex-vivo PBMC were fixed with the FOXP3 fixation/permeabilization kit (eBisocience, San Diego, CA, USA) and frozen in RPMI 1640 medium supplemented with 20% FBS and 10% DMSO and stored at -80°C. For immunophenotyping isolated PBMCs were stained with CD4-PE (SK3; BD Bioscience, San Diego, CA, USA), CD25-APC (M-A251; BD Bioscience, San Diego, CA, USA) and FOXP3-PE (PCH101; eBioscience, San Diego, CA, USA), acquired on a FACSCalibur flow cytometer (BD Biosciences, San Diego, CA, USA) and data were analysed in a FlowJo software (Tree Star Inc., Ashland, OR, US). To assess the frequency of CD25^hi^FOXP3^+^ Tregs, ex-vivo PBMC were fixed with the FOXP3 fixation/permeabilization kit (eBisocience, San Diego, CA, USA) and frozen in RPMI 1640 medium supplemented with 20% FBS and 10% DMSO and stored at -80°C. For immunophenotyping isolated PBMCs were stained with CD4-PE/Cy7 (SK3; BD Biosciences, San Diego, CA, USA), CD25-PE (2A3; BD Biosciences, San Diego, CA, USA) and FOXP3-APC (PCH101; eBioscience, San Diego, CA, USA), cells were acquired on FACSCanto II flow cytometer (BD Biosciences, San Diego, CA, USA) and analysed in FlowJo software (Tree Star Inc., Ashland, OR, US) using Boolean combination gates.

### Cytokines assays

Cytokines were measured from supernatants using Luminex 100 IS System (Invitrogen, Carlsbad, CA, USA) and commercially available beads and standards from BioSource (Bleiswijk, Netherlands) for interferon-gamma (IFN-γ), interleukin-5 (IL-5), IL-10, IL-13 and IL-17 and tumor necrosis factor (TNF). Beads were titrated for optimal dilution and used according to manufacturer’s instructions.

### Statistical analysis

Data analysis was performed using IBM SPSS Statistics version 20 for Windows (IBM Corp., Armonk, USA).

Differences between groups were determined by the Fisher’s exact test for sex, by Mann-Whitney U test for *S*. *haematobium* infection intensity and by the independent student’s T test for age and haematological parameters.

Cytokine concentrations in response to stimulation were corrected for spontaneous cytokine production by subtracting responses of unstimulated medium wells to obtain net cytokine responses, with negatives values set to half of the lowest value detected per given cytokine.

To avoid type I and type II errors in multiple testing, immunological parameters were reduced by principal-components analysis (PCA). First, R v2.15.1 Development Core Team software (R Foundation for Statistical Computing, Vienna, Austria, 2012, http://www.R-project.org) was used to estimate Box-Cox transformation parameter for each cytokine to increase normality of the data. Principal Component Analysis with Varimax rotation was used on all data points simultaneously (i.e. stimuli AWA/SEA/BCG; total and Treg-depleted PBMC; pre- and post-treatment time-points) to reduce the data into a smaller number of uncorrelated variables. Rotation converged in 3 iterations and principal components (PC) with eigenvalues greater than one were selected. Differences in PC scores between pre- and post-treatment and Treg-depleted and total PBMC were tested with the Wilcoxon matched pairs test. For all tests, statistical significance was considered at the 5% level.

## Results

### Elevated levels of CD4^+^CD25^hi^FOXP3^+^ Tregs in *S*. *haematobium*-infected schoolchildren

**Fig 1:**
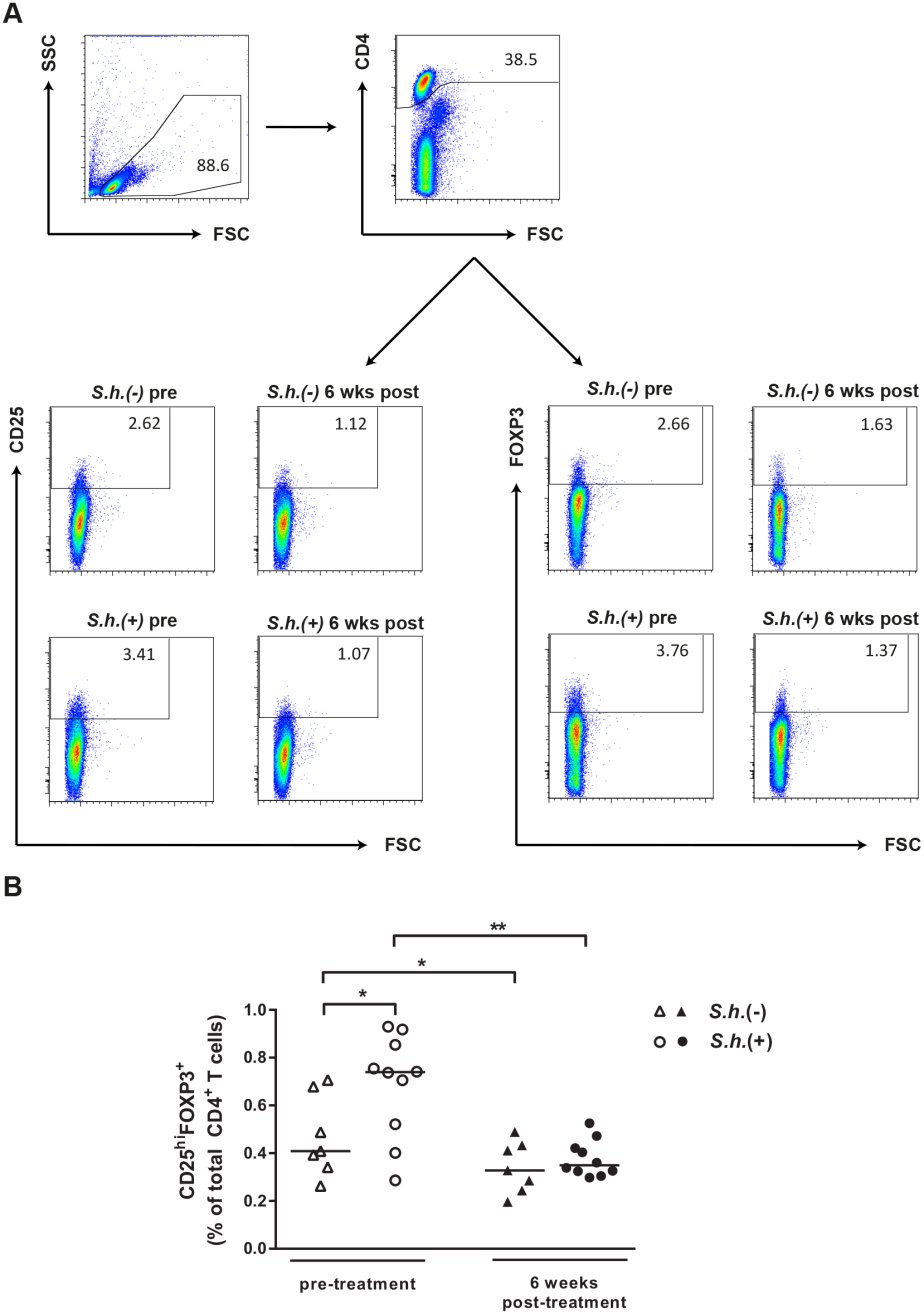
Increased frequency of CD4^+^CD25^hi^FOXP3^+^ Tregs during S. haematobium infection. Gating strategy for identification of CD4^+^CD25^hi^FOXP3^+^ Tregs (A). CD4 T cells were identified and Boolean gating combinations were used to determine proportions of CD4^+^CD25^hi^FOXP3^+^ Tregs (B). Differences between groups were tested with a Mann-Whitney U test and within groups with a Wilcoxon matched pairs test. Horizontal bars represent median. * p< 0.05, ** p< 0.01.

To investigate whether *S*. *haematobium* infection affects the frequency of peripheral blood Tregs we compared circulating CD4^+^CD25^hi^FOXP3^+^ Tregs from infected and uninfected children by flow cytometry. Gating strategy for identification of CD4^+^CD25^hi^FOXP3^+^ Tregs is shown in Fig 1A. We found that frequencies of FOXP3^+^ Tregs were significantly higher in infected children compared to uninfected children (Fig 1B). Importantly, six weeks after praziquantel treatment Treg frequencies were reduced by half to frequencies comparable to the uninfected control group. Over the same six weeks period, there was also a slight but consistent decrease in the Treg frequencies in the uninfected group.

### Proliferation and cytokine production in response to schistosome-specific and non-specific antigens in *S*. *haematobium*-infected schoolchildren at pre-treatment and 6 weeks post-treatment

**Fig 2:**
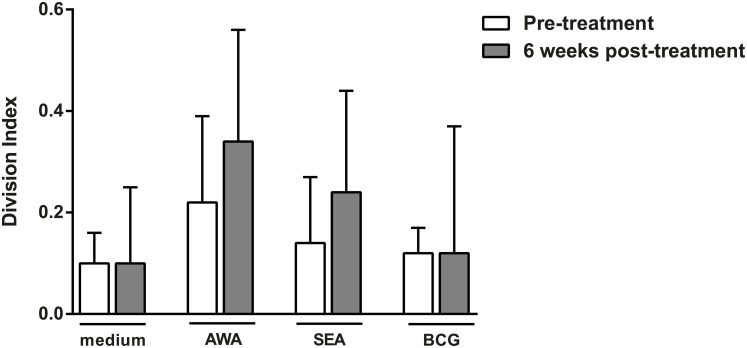
Proliferative responses to schistosome specific and non-specific antigens. CFSE-labelled total PBMC from *S*. *haematobium* infected children pre- and 6 weeks post-treatment were left unstimulated (medium), or stimulated with *S*. *haematobium* adult worm antigen (AWA) and soluble egg antigen (SEA) and Bacillus Calmette–Guérin (BCG). After 4 days of culture cells were fixed, cryopreserved and after thawing CFSE division was analyzed for CD4^+^CD25^hi^ T cells by flow cytometry. Results are shown as median with IQR. Differences between pre-treatment and 6 weeks post-treatment responses were tested with a Wilcoxon matched pairs test.

**Fig 3:**
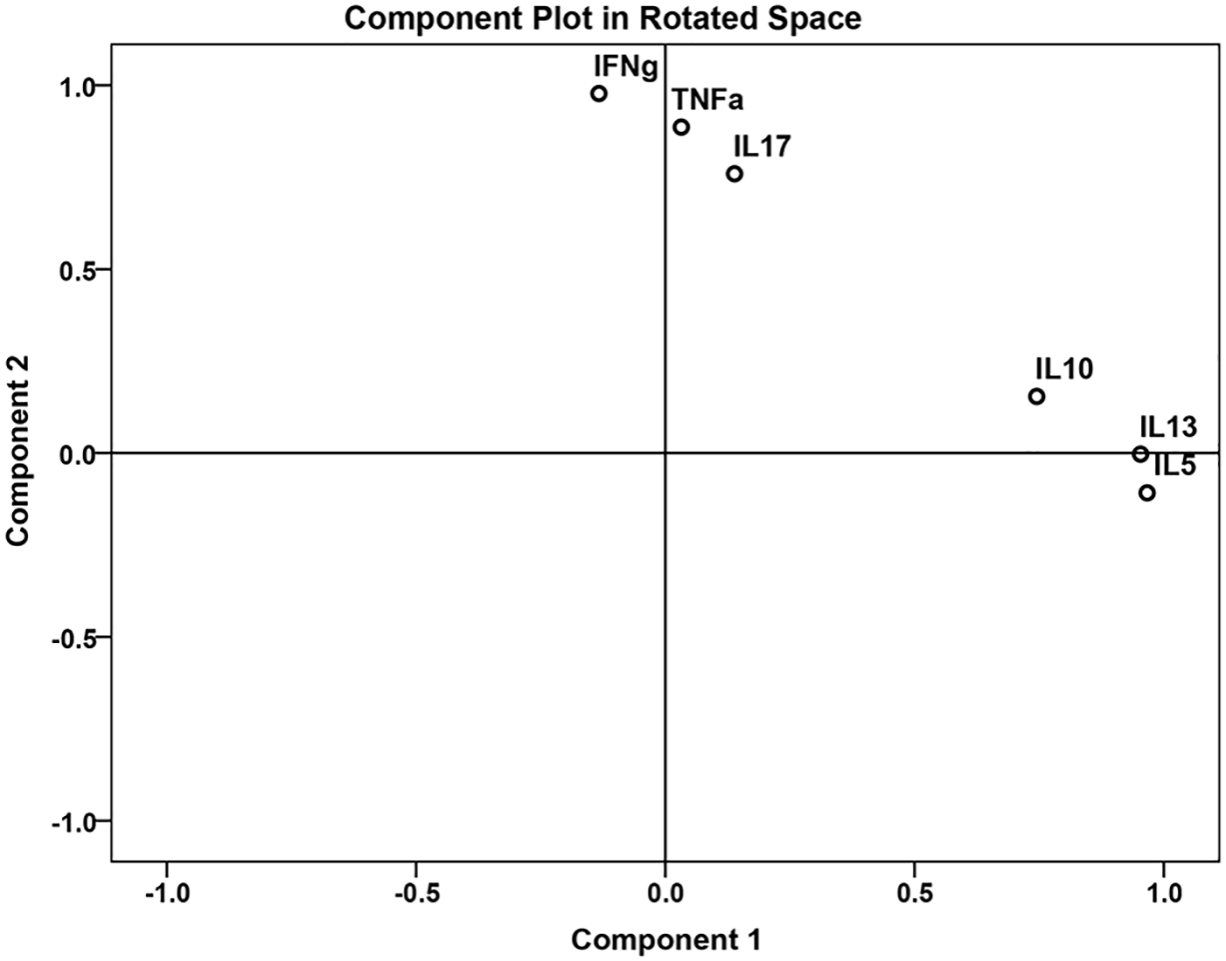
Principal component analysis (PCA) of cytokine responses to schistosome specific and non-specific antigens. Two distinct principal components were identified: principal component 1 (PC1) which reflects regulatory and Th2-polarized cytokine responses due to its positive loading with IL-5, IL-10 and IL-13 responses; and principal component 2 (PC2) which reflects pro-inflammatory and Th1-polarized cytokine responses due to its positive loading with IFN-γ, IL-17 and TNF.

Next, we assessed the effect of anthelmintic treatment on cell proliferation and cytokine production in response to stimulation with schistosome-specific antigens SEA and AWA and a non-specific antigen BCG. Proliferation was determined by calculating the division index on the basis of the dilution of CFSE in CD4^+^CD25^hi^ T cells. There were no significant differences in proliferation between pre-treatment and six weeks post-treatment responses (medium p = 0.397, AWA p = 0.188, SEA = 0.454 and BCG = 0.271) (Fig 2). Cytokine production on the other hand significantly changed between pre-treatment and 6 weeks post-treatment; raw cytokine values are shown in S1 Table. We applied Principal Component Analysis (PCA) in order to provide a more global assessment of the effect of schistosome infection on responses to not only SEA and AWA stimulation but also to the non-Ag specific stimulant BCG. Two distinct principal components were identified (Fig 3 and Table 1) which captured 73.7% of variance in our dataset: principal component 1 (PC1) which reflects regulatory and Th2-polarized cytokine responses due to its positive loading with IL-5, IL-10 and IL-13 responses (and accounted for 40% of the total variance in the data); and principal component 2 (PC2) which reflects pro-inflammatory and Th1-polarized cytokine responses due to its positive loading with IFN-γ, IL-17 and TNF (and accounted for 33.7% of total variance in the data. We saw a significant increase in both PC1 and PC2 following treatment compared to baseline values (Table 2).

**Table 1:** Description of principal components.

	Principal Component	
	1	2
IL-5	**0.897**	-0.250
IL-10	**0.774**	0.076
IL-13	**0.926**	-0.129
IL-17	0.080	**0.664**
IFN-γ	-0.351	**0.853**
TNF	-0.098	**0.875**

Arbitrary values indicate the relative loading of each cytokine response towards each principal component. Strong positive loadings (>0.500) are indicated in bold. IL, interleukin; IFN, interferon; TNF, tumor necrosis factor.

**Table 2:** Changes in cytokine production in response to antigens pre- to post-treatment.

Component	Time-point	Mean Rank	p value
1	pre-treatment	27.04	0.004
	post-treatment	41.14	
2	pre-treatment	31.41	0.021
	post-treatment	38.07	

### Enhanced T cell proliferation in T cells from schistosome-infected children after Treg depletion

To study the suppressive effect of Tregs on proliferation and cytokine responses, CD4^+^CD25^hi^ T cells were depleted from PBMC by magnetic beads. The CD4^+^CD25^hi^ population decreased by 45%, p = 0.0073 of which a representative example is shown in S1B Fig.

**Fig 4:**
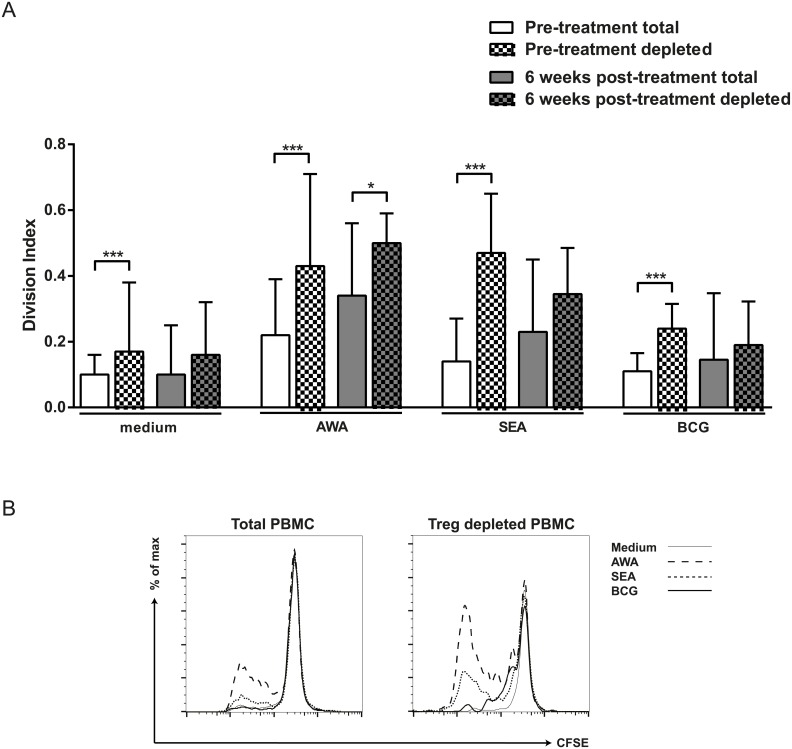
Effect of Treg depletion on proliferative responses to schistosome specific and non-specific antigens. CFSE-labeled total or CD4^+^CD25^hi^FOXP3^+^ depleted PBMC from *S*. *haematobium* infected children pre- and 6 weeks post-treatment were left unstimulated (medium), or stimulated with *S*. *haematobium* adult worm antigen (AWA) and soluble egg antigen (SEA) and Bacillus Calmette–Guérin (BCG). After 4 days of culture cells were fixed, cryopreserved and after thawing CFSE division was analyzed for CD4^+^CD25^hi^ T cells by flow cytometry. Results are shown as median with IQR (A). Differences between total and depleted PBMC were tested with a Wilcoxon matched pairs test. * p< 0.05, ** p< 0.01. Representative plot of CFSE staining illustrating proliferation of total or Treg depleted PBMC (B).

Depletion of Tregs at pre-treatment resulted in enhanced spontaneous proliferation (medium condition) as well as in enhanced proliferation to specific schistosomal antigens AWA and SEA and to vaccine antigen BCG (Fig 4A). At 6 weeks after anthelmintic treatment Treg depletion resulted in significant increase in proliferation in response to AWA only (Fig 4A). A typical plot of CFSE staining showing the effect induced by depletion of Tregs (Fig 4B).

### Increased cytokine responses following Treg depletion

Next, we investigated the capacity of Tregs to suppress cytokine responses by evaluating the effect of Treg depletion on principal component 1 (IL-5, IL-10 and IL-13) and principal component 2 (IFN-γ, IL-17 and TNF). We found that Treg depletion at pre-treatment resulted in increased values of both PC1 and PC2 in the infected individuals, and similarly at post-treatment, the depletion of Tregs resulted in an increase in the values of both PC1 and PC2 in the now infection free schoolchildren (Table 3).

**Table 3:** Changes in cytokine production in response to antigens in total and Treg depleted PBMC at pre- and post-treatment.

Component	Time-point	Total/Treg depleted PBMCs	Mean Rank	p value
1	pre-tx	total	32.78	0.002
		depleted	42.36	
	post-tx	total	33.58	0.002
		depleted	39.38	
2	pre-tx	total	28.09	<0.001
		depleted	43.36	
	post-tx	total	32.35	<0.001
		depleted	39.41	

tx, treatment.

## Discussion

Down-regulation of immune responses has been attributed to a strong immunomodulatory network of regulatory cells induced by schistosomes [25,26]. Here we provide evidence that human *Schistosoma* infection is associated with increased FOXP3^+^ regulatory T cells that play a significant role in controlling Th1 and Th2 responses. This finding is consistent with reports of increased numbers of FOXP3^+^ Tregs in peripheral blood from children 8–13 years old with active schistosomiasis [27] as well as other helminth infections including lymphatic filariasis [14,28]. Moreover, the reduction in the number of FOXP3^+^ Treg after treatment is in line with reports showing that drug induced clearance of *Shistosoma* parasites reduces Treg numbers defined only as CD4^+^CD25^hi^ [18].

A much smaller in magnitude, yet statistically significant decrease was observed in the frequency of Tregs in the uninfected control group, which indicates that there were additional factors that affected the measured frequency of the CD4^+^CD25^hi^FOXP3^+^ cells; this could include technical or environmental changes such as seasonal effects, that might be associated with longitudinal studies. However, as both sampling time-points occurred during the long rainy season changes in Treg frequency in the control group are less likely to reflect seasonal changes and other factors such as exposure to concomitant infections could play a role.

In order to obtain a global assessment of the effect of *S*. *haematobium* infection on Th1, Th2, regulatory and pro-inflammatory cytokine responses we applied PCA analysis. This allowed us to summarize the various responses into two principal components [29]. Principal component 1 (PC1) reflected regulatory and Th2-polarized cytokine responses due to its positive loading with IL-5, IL-10 and IL-13, responses commonly associated with chronic schistosome infections. Principal component 2 (PC2) reflected pro-inflammatory and Th1-polarized cytokine responses due to its positive loading with IFN-γ, IL-17 and TNF, responses more commonly associated with acute schistosome infection or bacterial infections such as tuberculosis. We show that *S*. *haematobium* infection is associated with hypo-responsiveness as demonstrated by increases in cytokine production represented by both PC1 and PC2 following treatment of schistosomiasis with praziquantel. T cell division was also assessed, but despite the consistently higher proliferation to all stimuli tested, at post treatment, the change was not statistically significant. These data indicate that the increased frequency of CD4^+^CD25^hi^FOXP3^+^ Tregs during schistosome infection may be associated with poor cytokine responsiveness.

To assess the functional capacity of the regulatory T cells, a field applicable method was used which consists of the depletion of regulatory T cells from PBMC to assess their effect on cytokine production or proliferation. The data show that depletion of Tregs is associated with increased cytokine production, of both PC1 and PC2 which means that both Th2/regulatory and Th1/pro-inflammatory cytokine production improves. This is the case at both pre-treatment and post-treatment time-points, although the increase appears to be stronger at pre-treatment. Altogether, this would suggest that even though regulatory T cell numbers change with infection, their functional capacity to supress cytokine production remains.

Furthermore we evaluated the effect of Treg depletion on cell proliferation. While cytokine responses were similarly affected at both pre-and post-treatment, proliferative responses were predominantly affected by Treg depletion in infected individuals at pre-treatment only. These data could be explained if the ability of Tregs to suppress proliferation would be distinct from suppressive mechanisms required for inhibiting cytokine production, for example Tregs only supress PBMC proliferation in a higher Treg:responder ratio as seen during active infection. The removal of *S*. *haematobium* infection could then affect the Treg function partially. Tregs are thought to exert their function via a number of different mechanisms including IL-10 and/or TGF-β production, IL-2 consumption, or cell-cell contact where inhibitory molecules such as CTLA-4 and PD-1 are key [30,31]. A recent study suggests a unique role for microRNAs in suppression of T cell proliferation by Tregs [32]. Future studies are needed to delineate how Tregs exert their suppressive role during the course of schistosome infection and furthermore the difference in mechanism between suppression of proliferation versus effector cytokine production. Additional alternative mechanism, such as T cell anergy due to increased expression of the E3 ubiquitin ligase GRAIL (gene related to anergy in lymphocytes) which has been shown in a mouse model to be linked to Th2 cell hypo-responsiveness could also play a role and should also be investigated in future studies [33]. The role of inflammation in the induction and maintenance of Treg cells should likewise be considered as *Shistosoma* infection is a chronic inflammatory disorder and Tregs protect the human host against excessive inflammation, thus increased Treg numbers during schistosomisis may be a responses against inflammation rather than directly induced by the parasites [34].

Alternatively, recently described regulatory CD8^+^ T cells which likewise produce IL-10, may also in part contribute to the differences observed [35,36]. CD25^hi^ cell depletion will in addition to depleting CD4^+^CD25^hi^FOXP3^+^ T cells also deplete the CD8^+^CD25^hi^FOXP3^+^ T cell population and therefore future studies are needed to re-assess the relative contributions of these different subsets. Moreover studies with more extensive panels of markers associated with suppressive T cell functions are necessary as FOXP3 expression has been shown to be transiently up-regulated on activated CD4^+^ T cells [37].

Finally, the concomitant role of the different regulatory cells, including in addition to Tregs, regulatory B cells [38] and regulatory monocytes/macrophages [39,40] and their relative contribution to the suppressive activities observed need to be further investigated.

In summary, this study shows that infection with *S*. *haematobium* is associated with alterations of the frequency and activity of CD4^+^CD25^hi^FOXP3^+^ regulatory T cells and that these in turn affect proliferation and global cytokine responses. These data indicate that the functional activity of regulatory T cells needs to be taken into consideration when studies consider co-infections, treatment or vaccine responses in areas where helminths are prevalent.

## Supporting Information

S1 Fig Treg depletion. Representative examples of the depletion of the CD4^+^CD25^hi^FOXP3^+^ population (A). CD4^+^CD25^hi^ T cells were depleted by magnetic bead separation (B).
(EPS)

S1 Table Raw cytokine values following antigen stimulation in total and Treg depleted PBMC at pre- and post-treatment. (DOCX)
