# Supplementary material for: CD4+CD25hiFOXP3+ Regulatory T Cells and Cytokine Responses in Human Schistosomiasis before and after Treatment with Praziquantel
Source: PLoS Negl Trop Dis. 2015 Aug 20;9(8):e0003995. doi: 10.1371/journal.pntd.0003995 (PMC4546370; doi:10.1371/journal.pntd.0003995)
Supplement: S1 Table — (DOCX) [file pntd.0003995.s002.docx]

| Cytokine | Stimulus | Pre-treatment | |  | 6 weeks post-treatment | |
| --- | --- | --- | --- | --- | --- | --- |
|  |  | Total (median (IQR)) | Treg depleted (median (IQR)) |  | Total (median (IQR)) | Treg depleted (median (IQR)) |
| IL-5 | medium | 28.97 (10.61 - 91.81) | 29.67 (9.75 - 90.39) |  | 69.40 (39.08 - 154.05) | 43.31 (16.58 - 149.90) |
|  | AWA | 475.86 (173.95 - 774.07) | 372.44 (166.76 - 827.32) |  | 721.88 (373.64 - 1185.76) | 745.63 (337.48 - 1112.00) |
|  | SEA | 127.50 (62.61 - 241.38) | 156.14 (115.71 - 615.18) |  | 184.83 (89.09 - 469.41) | 257.78 (102.46 - 440.22) |
|  | BCG | 4.96 (3.00 - 13.63) | 4.48 (3.00 - 14.75) |  | 14.11 (3.00 - 36.76) | 12.67 (3.23 - 38.88) |
|  |  |  |  |  |  |  |
| IL-13 | medium | 101.43 (14.34 - 141.82) | 88.75 (33.64 - 198.27) |  | 142.17 (47.78 - 341.57) | 163.38 (47.78 - 363.11) |
|  | AWA | 570.86 (339.74 - 1155.67) | 798.86 (484.03 - 1237.20) |  | 1047.52 (657.20 - 1984.41) | 1359.63 (714.84 - 2126.23) |
|  | SEA | 254.60 (135.16 - 467.21) | 442.24 (280.61 - 946.45) |  | 522.26 (150.21 - 897.83) | 728.83 (277.69 - 1357.21) |
|  | BCG | 32.70 (10.06 - 82.64) | 85.59 (18.02 - 146.92) |  | 83.64 (17.36 - 217.83) | 98.46 (28.14 - 255.57) |
|  |  |  |  |  |  |  |
| IFN-γ | medium | 10.01 (5.00 - 28.82) | 28.82 (14.34 - 58.51) |  | 11.37 (5.00 - 30.13) | 13.36 (5.00 - 66.35) |
|  | AWA | 13.52 (5.00 - 36.19) | 43.64 (12.02 - 106.81) |  | 6.98 (5.00 - 30.10) | 26.69 (5.53 - 91.46) |
|  | SEA | 8.18 (5.00 - 24.08) | 30.03 (10.25 - 102.82) |  | 6.95 (5.00 - 29.77) | 41.74 (9.56 - 75.56) |
|  | BCG | 266.25 (84.88 - 911.19) | 728.69 (400.98 - 2457.31) |  | 377.74 (200.41 - 1466.34) | 1262.53 (453.56 - 3996.58) |
|  |  |  |  |  |  |  |
| IL-10 | medium | 15.00 (15.00 - 28.61) | 15.00 (15.00 - 17.63) |  | 15.00 (15.00 - 42.94) | 15.00 (15.00 - 32.71) |
|  | AWA | 199.99 (92.99 - 306.86) | 199.40 (110.98 - 387.70) |  | 288.56 (120.15 - 564.33) | 444.80 (212.37 - 645.22) |
|  | SEA | 132.81 (65.02 - 216.76) | 164.55 (84.82 - 282.93) |  | 186.01 (77.29 - 373.99) | 244.98 (86.71 - 348.82) |
|  | BCG | 63.48 (15.00 - 117.97) | 63.07 (15.86 - 115.49) |  | 135.74 (62.28 - 196.93) | 130.88 (51.06 - 270.97) |
|  |  |  |  |  |  |  |
| IL-17 | medium | 10.00 (10.00 - 19.15) | 10.00 (10.00 - 11.35) |  | 10.00 (10.00 - 13.63) | 10.78 (10.00 - 41.59) |
|  | AWA | 10.00 (10.00 - 31.93) | 25.59 (10.00 - 60.03) |  | 18.09 (10.00 - 41.01) | 27.41 (12.39 - 82.30) |
|  | SEA | 11.52 (10.00 - 38.83) | 19.11 (10.00 - 44.83) |  | 10.00 (10.00 - 37.55) | 38.63 (11.51 - 64.09) |
|  | BCG | 39.70 (10.00 - 74.94) | 36.89 (20.00 - 174.11) |  | 65.54 (20.62 - 118.98) | 109.85 (22.72 - 155.26) |
|  |  |  |  |  |  |  |
| TNF | medium | 46.29 (14.33 - 125.45) | 42.53 (15.66 - 178.20) |  | 98.50 (33.23 - 233.55) | 101.56 (25.05 - 256.81) |
|  | AWA | 154.54 (85.24 - 231.39) | 330.86 (167.01 - 529.55) |  | 224.74 (101.72 - 335.88) | 306.40 (122.04 - 582.24) |
|  | SEA | 119.93 (30.84 - 265.32) | 201.29 (44.71 - 480.41) |  | 94.31 (42.38 - 261.80) | 172.38 (66.79 - 366.74) |
|  | BCG | 537.67 (183.00 - 1268.92) | 1708.70 (738.50 - 5445.60) |  | 1024.85 (422.46 - 2013.62) | 1641.89 (675.63 - 4619.88) |

IL, interleukin; IFN, interferon; TNF, tumor necrosis factor; AWA, adult worm antigen; SEA, soluble egg antigen; BCG, Bacillus Calmette–Guérin (BCG)
